# Self-regulated learning and academic success under digital inequality among Peruvian university students: the moderating role of institutional support

**DOI:** 10.3389/fpsyg.2026.1838281

**Published:** 2026-08-18

**Authors:** Mónica Beatriz Juárez López, Walter Stalin Gil Quevedo, Abraham William García Chapoñan, Elena Luida Laos Fernández

**Affiliations:** Facultad de Educación, Universidad Nacional José Faustino Sánchez Carrión, Huacho, Peru

**Keywords:** academic success, digital inequality, educational psychology, higher education, institutional support, Peru, self-regulated learning, structural equation modeling

## Abstract

Academic success in higher education is influenced by both psychological competencies and contextual conditions, particularly in environments characterized by unequal access to digital resources. Although previous studies have examined self-regulated learning and digital inequality separately, limited evidence has integrated these constructs within a single analytical framework in Latin American higher education. This study examined the relationships among digital inequality, self-regulated learning, institutional support, and academic success in a sample of 742 undergraduate students from a public university in Peru. A quantitative cross-sectional design was employed, and the proposed theoretical model was evaluated using covariance-based structural equation modeling (CB-SEM). The findings revealed significant associations among the study variables. Self-regulated learning showed the strongest positive association with academic success and partially mediated the relationship between digital inequality and academic success. Institutional support moderated the associations between digital inequality and academic success, as well as between self-regulated learning and academic success, suggesting that supportive educational environments may buffer the adverse effects of limited digital access. Although the cross-sectional nature of the study precludes causal inference, the findings contribute to understanding how psychological and contextual factors jointly relate to academic success in resource-constrained higher education settings. These results provide evidence to support institutional strategies aimed at strengthening students’ self-regulated learning competencies while reducing digital inequalities.

## 1 Introduction

Academic success in higher education has traditionally been explained through cognitive ability, prior academic achievement, and socioeconomic background. More recently, educational psychology has shifted toward multidimensional frameworks recognizing that academic outcomes emerge from the interaction between individual psychological competencies and contextual learning environments (Zimmerman, 2002; Panadero, 2017). Within these frameworks, self-regulated learning has become one of the most influential constructs for understanding how students actively manage their learning processes.

Self-regulated learning refers to learners’ capacity to establish academic goals, monitor their cognitive and motivational processes, regulate learning strategies, and evaluate their own progress (Zimmerman, 2002). Rather than representing a stable personal trait, contemporary perspectives conceptualize self-regulated learning as a dynamic process that evolves according to academic experiences and environmental conditions (Schunk and Greene, 2018). Consequently, students’ ability to self-regulate may vary depending on the availability of educational resources and institutional support (Broadbent and Poon, 2015; Cleary and Zimmerman, 2012; Dignath et al., 2008; Hodges et al., 2020; Jansen et al., 2019).

At the same time, the growing digitalization of higher education has increased attention toward digital inequality. Current evidence indicates that digital inequality extends beyond physical access to technological devices and internet connectivity. It also includes digital competencies, patterns of technology use, and individuals’ capacity to transform digital resources into meaningful educational opportunities (van Dijk, 2020). Therefore, digital inequality should be understood as a multidimensional construct reflecting differences in digital access, digital skills, quality of technology use, and educational outcomes derived from technology.

From this perspective, digital inequality and self-regulated learning should not be viewed as completely independent phenomena. Limited digital resources may constrain opportunities to develop effective self-regulation strategies, whereas students with stronger self-regulatory competencies may better adapt to restricted digital environments by optimizing the resources available to them. This reciprocal relationship has received increasing attention in educational psychology, although empirical evidence remains limited, particularly in developing countries.

Institutional support represents another contextual factor that may influence these relationships. Previous studies have shown that students’ perceptions of teaching quality, academic advising, technological infrastructure, and institutional responsiveness contribute significantly to academic engagement and performance (Kuh et al., 2008). Rather than functioning independently, institutional support may strengthen the effectiveness of self-regulated learning while simultaneously reducing some of the negative consequences associated with digital inequality.

Despite growing research in this field, important gaps remain. First, relatively few studies have simultaneously examined digital inequality, self-regulated learning, institutional support, and academic success within a single theoretical model. Second, empirical evidence from Latin American higher education remains limited despite substantial inequalities in digital infrastructure. Third, although structural equation modeling has become increasingly common in educational psychology, few studies have evaluated the simultaneous direct, indirect, and moderating relationships among these constructs while acknowledging the limitations imposed by cross-sectional data.

Peru provides an appropriate context for examining these relationships. Although university enrollment has expanded considerably during recent decades, substantial disparities remain regarding access to digital technologies, institutional resources, and educational opportunities, particularly among public universities outside metropolitan regions (OECD, 2020). These structural conditions make it possible to examine how individual psychological competencies and institutional factors are associated with academic success under conditions of digital inequality.

Accordingly, the present study proposes a theoretical model examining the associations among digital inequality, self-regulated learning, institutional support, and academic success in Peruvian university students. Given the cross-sectional design, the proposed relationships should be interpreted as theoretical associations rather than causal effects.

The specific objectives of this study were:

1. To examine the association between digital inequality and academic success.
2. To evaluate the relationship between self-regulated learning and academic success.
3. To examine the association between digital inequality and self-regulated learning.
4. To assess whether self-regulated learning statistically mediates the association between digital inequality and academic success.
5. To evaluate whether perceived institutional support moderates the associations among the study variables.

Based on previous literature, the following hypotheses were proposed:

- H1: Digital inequality is negatively associated with academic success.
- H2: Self-regulated learning is positively associated with academic success.
- H3: Digital inequality is negatively associated with self-regulated learning.
- H4: Self-regulated learning statistically mediates the association between digital inequality and academic success.
- H5: Perceived institutional support is positively associated with academic success.
- H6: Perceived institutional support moderates the association between digital inequality and academic success.
- H7: Perceived institutional support moderates the association between self-regulated learning and academic success.

## 2 Materials and methods

### 2.1 Study design

This study adopted a quantitative, observational, cross-sectional design to examine the relationships among digital inequality, self-regulated learning, perceived institutional support, and academic success among undergraduate students enrolled in a public university in Peru.

Because the study was based on data collected at a single point in time, the proposed structural relationships should be interpreted as statistical associations rather than causal effects. Although structural equation modeling allows the simultaneous estimation of complex direct, indirect, and moderating relationships, temporal precedence cannot be established in cross-sectional research. Therefore, mediation analyses should be understood as theory-driven statistical models rather than evidence of causal mechanisms.

A covariance-based structural equation modeling (CB-SEM) approach was selected because the proposed theoretical model was developed from established educational psychology theories and the primary objective was theory testing rather than prediction. CB-SEM is particularly appropriate when evaluating latent psychological constructs measured through validated multi-item scales and when assessing overall model fit using global goodness-of-fit indices (Kline, 2016; Hair et al., 2019).

### 2.2 Participants

The study included 742 undergraduate students enrolled at Universidad Nacional José Faustino Sánchez Carrión, a public university located in Huacho, Peru.

Participants were recruited using convenience sampling because institutional regulations did not permit access to complete enrollment lists for probabilistic selection. Although this sampling strategy limits statistical generalizability, it is frequently employed in higher education research where complete sampling frames are unavailable.

Eligible participants met the following inclusion criteria:

- being officially enrolled as undergraduate students during the second academic semester of 2025;
- having completed at least one semester involving digital or blended learning activities;
- being at least 18 years of age;
- voluntarily providing informed consent.

Students who submitted incomplete questionnaires exceeding 10% missing responses were excluded from the analyses.

The final sample represented students from several academic disciplines, including engineering, social sciences, education, business administration, and health sciences.

The demographic characteristics were as follows:

- Mean age = 21.4 years (SD = 3.2)
- Age range = 18–33 years
- Female = 54%
- Male = 46%

Including students from multiple academic programs increased the heterogeneity of the sample and reduced the likelihood that the observed relationships were discipline-specific.

Nevertheless, because participants were recruited from a single institution, the findings should be interpreted cautiously and should not be generalized to all Peruvian university students.

### 2.3 Measures

All constructs were assessed using previously validated instruments translated into Spanish through a translation and back-translation procedure following international recommendations for cross-cultural adaptation.

Before data collection, a pilot study involving 35 undergraduate students was conducted to evaluate item clarity and cultural appropriateness. Minor wording adjustments were introduced to improve readability while preserving the conceptual meaning of the original instruments.

Construct validity was subsequently examined through confirmatory factor analysis (CFA) using covariance-based SEM.

Reliability was evaluated using Cronbach’s alpha and Composite Reliability (CR). Convergent validity was assessed through Average Variance Extracted (AVE), whereas discriminant validity was evaluated using both the Fornell–Larcker criterion and the Heterotrait–Monotrait Ratio (HTMT), which has been recommended as a more sensitive indicator of discriminant validity in contemporary SEM research.

#### 2.3.1 Self-regulated learning

Self-regulated learning was assessed using an adapted Spanish version of the Motivated Strategies for Learning Questionnaire (MSLQ) originally developed by Pintrich et al. (1991).

The instrument measures three complementary dimensions:

- cognitive learning strategies;
- metacognitive regulation;
- effort regulation.

Participants responded using a five-point Likert scale ranging from 1 (strongly disagree) to 5 (strongly agree).

Although seven-point scales may provide greater measurement sensitivity, the five-point format was retained because it has demonstrated satisfactory psychometric performance in previous studies conducted in Latin American educational contexts and facilitated respondents’ comprehension.

#### 2.3.2 Digital inequality

Digital inequality was conceptualized as a multidimensional construct rather than merely the absence of technological access.

Accordingly, the measurement instrument incorporated four complementary dimensions:

- access to digital devices;
- internet connectivity quality;
- digital competencies for academic purposes;
- meaningful educational use of digital technologies.

Items were adapted from previous studies addressing the multidimensional nature of digital inequality (van Dijk, 2020; Means and Neisler, 2021).

Rather than assigning arbitrary weights to the different dimensions, all indicators were modeled as reflective measures of a latent construct representing perceived digital inequality.

Higher scores indicated greater perceived digital inequality.

#### 2.3.3 Institutional support

Perceived institutional support was measured using students’ subjective evaluations of the educational environment, including perceived teaching quality, academic advising, technological infrastructure, and institutional responsiveness.

The construct represents students’ perceptions of institutional support rather than objective institutional characteristics. Consequently, findings should be interpreted as reflecting perceived institutional experiences.

#### 2.3.4 Academic success

Academic success was operationalized using two complementary indicators:

- self-reported cumulative grade point average (GPA);
- perceived academic performance.

Although official institutional academic records would provide a more objective assessment of academic achievement, access to such records was restricted because of institutional confidentiality policies.

Therefore, self-reported GPA was used as a proxy measure. Previous research has shown that self-reported GPA is generally highly correlated with official academic records; however, potential reporting bias cannot be completely excluded.

### 2.4 Procedure

Data collection was conducted during the second academic semester of 2025 using an online questionnaire administered through the university’s institutional learning platform.

Before completing the survey, all participants received detailed information regarding the objectives of the study, voluntary participation, confidentiality, anonymity, and the right to withdraw at any stage without academic consequences. Electronic informed consent was obtained before participants accessed the questionnaire.

The average completion time ranged between 15 and 20 min.

To minimize common method bias (CMB), several procedural strategies were implemented following recommendations by Podsakoff et al. (2003). These included:

- guaranteeing complete anonymity of responses;
- emphasizing that there were no correct or incorrect answers;
- randomizing the order of questionnaire items;
- separating items measuring predictors and outcome variables;
- using different verbal anchors across measurement sections.

Although these procedures reduce the likelihood of common method variance, the exclusive reliance on self-reported measures remains an inherent limitation of the study.

### 2.5 Data analysis

Statistical analyses were performed using IBM SPSS Statistics 29 and IBM SPSS AMOS 29.

Because the objective of the study was to test a theory-driven model derived from established educational psychology frameworks, covariance-based structural equation modeling (CB-SEM) was selected instead of partial least squares SEM (PLS-SEM). This analytical approach allows simultaneous estimation of measurement and structural models while providing global goodness-of-fit indices appropriate for confirmatory research.

#### 2.5.1 Preliminary data screening

Prior to structural analyses, the dataset was examined for:

- missing values;
- univariate normality;
- multivariate normality;
- multicollinearity;
- multivariate outliers.

Missing values accounted for less than 2% of the dataset and were handled using the Expectation-Maximization (EM) algorithm.

Univariate normality was evaluated through skewness and kurtosis coefficients, adopting acceptable thresholds of ±2.

Multicollinearity was examined using Variance Inflation Factors (VIF), with all values remaining below 3.0, indicating no serious collinearity concerns.

Potential multivariate outliers were evaluated using Mahalanobis distance. Cases identified as extreme observations were carefully inspected; however, none were excluded because their inclusion did not materially affect model estimation.

#### 2.5.2 Measurement model evaluation

The psychometric properties of the measurement model were evaluated using Confirmatory Factor Analysis (CFA).

Reliability was assessed through:

- Cronbach’s alpha (α)
- Composite Reliability (CR)

Convergent validity was evaluated using the Average Variance Extracted (AVE), adopting the recommended threshold of AVE ≥ 0.50.

Discriminant validity was examined using two complementary procedures:

- Fornell–Larcker criterion
- Heterotrait–Monotrait Ratio (HTMT)

HTMT values below 0.85 were considered indicative of satisfactory discriminant validity, following recent SEM recommendations.

#### 2.5.3 Structural model evaluation

After establishing adequate measurement properties, the structural model was estimated.

The following statistics were examined:

- standardized path coefficients (β);
- standard errors;
- *p*-values;
- coefficients of determination (R^2^);
- effect sizes (f^2^);
- bootstrapped confidence intervals (5,000 resamples).

Because the proposed model included both mediation and moderation hypotheses, indirect effects were estimated using bias-corrected bootstrapping with 5,000 resamples.

Moderation analyses were conducted by estimating latent interaction terms within the CB-SEM framework.

#### 2.5.4 Model fit evaluation

Overall model fit was evaluated using multiple complementary indices:

**Table:** 

Fit index	Recommended value
χ^2^/df	<3.00
CFI	≥0.90
TLI	≥0.90
RMSEA	≤0.08
SRMR	≤0.08

Modification indices were examined after the initial CFA to identify localized areas of model misfit. However, only theoretically justifiable modifications were considered. No data-driven modifications that substantially altered the theoretical model were implemented.

### 2.6 Ethical considerations

This study was conducted in accordance with the ethical principles established in the Declaration of Helsinki.

Ethical approval was obtained from the Research Ethics Committee of Universidad Nacional José Faustino Sánchez Carrión before data collection.

Participation was entirely voluntary. All participants provided electronic informed consent after receiving detailed information regarding the objectives of the study, confidentiality of responses, data protection procedures, and their right to withdraw without consequences.

No personally identifiable information was collected, and all analyses were performed using anonymized datasets.

The data were used exclusively for scientific purposes and stored following institutional regulations on research integrity and confidentiality.

## 3 Results

### 3.1 Preliminary data screening

Prior to hypothesis testing, the dataset was examined for completeness, distributional assumptions, multicollinearity, and potential outliers.

Missing data represented less than 2% of the total observations and were handled using the Expectation-Maximization algorithm because the proportion of missing values was minimal and consistent with recommendations for SEM analyses.

Univariate normality was assessed through skewness and kurtosis coefficients. All observed values remained within the acceptable range (±2), supporting the use of Maximum Likelihood estimation.

Multicollinearity diagnostics indicated no evidence of problematic redundancy among predictor variables, with Variance Inflation Factors (VIF) below the recommended threshold of 3.0.

Potential multivariate outliers were evaluated using Mahalanobis distance. Although a small number of observations exceeded the critical value, inspection revealed no influential cases requiring exclusion, and all participants were retained in the final analyses.

Pearson correlation coefficients showed statistically significant associations among all study variables (Table 1). Digital inequality was negatively associated with both self-regulated learning and academic success, whereas perceived institutional support showed positive associations with self-regulated learning and academic success.

**TABLE 1:** Descriptive statistics and Pearson correlations among the study variables.

Variable	Mean	SD	1	2	3	4
1. Digital Inequality	2.85	0.74	–	–	–	–
2. Self-Regulated Learning	3.67	0.68	−0.34**			
3. Institutional Support	3.45	0.71	−0.21**	0.42**		
4. Academic Success	3.58	0.65	−0.28**	0.52**	0.39**	

Correlation coefficients represent Pearson’s *r*. Digital inequality scores were coded so that higher values indicate greater perceived digital inequality. **p < 0.01.

### 3.2 Measurement model

A confirmatory factor analysis (CFA) was conducted to evaluate the psychometric properties of the measurement model prior to estimating the structural relationships.

The results indicated satisfactory measurement performance across all latent constructs. Standardized factor loadings ranged from 0.69 to 0.88, exceeding the minimum recommended threshold of 0.60 and supporting adequate indicator reliability.

Composite Reliability (CR) values ranged from 0.86 to 0.93, demonstrating satisfactory internal consistency.

Average Variance Extracted (AVE) values exceeded the recommended threshold of 0.50 for all constructs, providing evidence of convergent validity.

Discriminant validity was evaluated using the Fornell–Larcker criterion. In addition, the Heterotrait–Monotrait Ratio (HTMT) was examined as a complementary indicator. HTMT values remained below the recommended threshold of 0.85, supporting satisfactory discriminant validity among the latent constructs.

#### 3.2.1 Reliability and convergent validity

The reliability analysis demonstrated satisfactory internal consistency across all latent constructs.

Cronbach’s alpha coefficients ranged between 0.83 and 0.91, while Composite Reliability values varied from 0.86 to 0.93, exceeding the recommended threshold of 0.70.

Similarly, Average Variance Extracted values ranged from 0.56 to 0.65, confirming that each construct explained more than half of the variance of its observed indicators.

Taken together, these findings provide evidence supporting the reliability and convergent validity of the measurement model as shown in Table 2.

**TABLE 2:** Measurement model: factor loadings, reliability, and validity.

Construct	Item loadings	CR	AVE
Self-regulated learning	0.71–0.88	0.93	0.65
Digital inequality	0.69–0.85	0.89	0.58
Institutional support	0.72–0.87	0.91	0.62
Academic success	0.70–0.84	0.86	0.56

CR, composite reliability; AVE, average variance extracted.

#### 3.2.2 Discriminant validity

Discriminant validity was first evaluated using the Fornell–Larcker criterion.

For every construct, the square root of the Average Variance Extracted exceeded its correlations with the remaining latent variables, indicating adequate discriminant validity.

These findings suggest that each construct captured a distinct conceptual domain despite the theoretically expected relationships among self-regulated learning, institutional support, digital inequality, and academic success.

#### 3.2.3 Model fit

The confirmatory factor analysis demonstrated satisfactory overall model fit.

The ratio between Chi-square and degrees of freedom was 2.41, indicating acceptable model parsimony.

Similarly, the Comparative Fit Index (CFI = 0.94) and Tucker-Lewis Index (TLI = 0.93) exceeded the recommended threshold of 0.90.

Absolute fit indices also supported model adequacy, with RMSEA (0.044) and SRMR (0.047) remaining well below the recommended cutoff of 0.08 as summarized in Table 3.

**TABLE 3:** Goodness-of-fit indices for the measurement model.

Fit index	Value	Recommended threshold
χ^2^/df	2.41	<3.00
CFI	0.94	≥0.90
TLI	0.93	≥0.90
RMSEA	0.044	≤0.08
SRMR	0.047	≤0.08

Taken together, these indices indicate that the measurement model adequately represents the covariance structure observed in the data.

### 3.3 Structural model

After confirming the adequacy of the measurement model, the structural model was estimated to evaluate the hypothesized associations among the latent constructs.

Standardized path coefficients, standard errors, and significance levels are presented in Table 4.

**TABLE 4:** Standardized structural path coefficients.

Hypothesis	Path	β	SE	p-value
H1	Digital Inequality → Academic Success	−0.28	0.05	<0.001
H2	Self-Regulated Learning → Academic Success	0.52	0.06	<0.001
H3	Digital Inequality → Self-Regulated Learning	−0.34	0.05	<0.001
H5	Institutional Support → Academic Success	0.29	0.05	<0.001

Overall, the structural model demonstrated statistically significant associations consistent with the proposed theoretical framework.

#### 3.3.1 Direct effects

The structural model revealed statistically significant associations between the principal study variables.

Digital inequality was negatively associated with academic success (β = –0.28, *p* < 0.001), indicating that students reporting greater digital inequality also tended to report lower academic success.

Self-regulated learning exhibited the strongest positive association with academic success (β = 0.52, *p* < 0.001), suggesting that students reporting higher levels of self-regulation also tended to report higher academic achievement.

Digital inequality was negatively associated with self-regulated learning (β = –0.34, *p* < 0.001), indicating that greater perceived digital inequality was related to lower levels of self-regulated learning.

Finally, perceived institutional support demonstrated a positive association with academic success (β = 0.29, *p* < 0.001).

Because the study employed a cross-sectional design, these findings should be interpreted as statistical associations rather than causal effects.

#### 3.3.2 Mediation analysis

The mediating role of self-regulated learning in the association between digital inequality and academic success was evaluated using a bias-corrected bootstrapping procedure with 5,000 resamples.

The indirect effect was statistically significant (β = –0.18, *p* < 0.001), with a 95% confidence interval ranging from –0.24 to –0.12, excluding zero.

These findings suggest that self-regulated learning statistically mediates the association between digital inequality and academic success. Specifically, students reporting greater digital inequality also tended to report lower levels of self-regulated learning, which, in turn, were associated with lower academic success.

Because the study employed a cross-sectional design, this mediation should be interpreted as a statistical indirect association rather than evidence of a temporal or causal mediation process.

#### 3.3.3 Moderation analysis

Moderation effects were estimated by incorporating latent interaction terms within the structural model.

The interaction between institutional support and digital inequality was statistically significant (β = 0.15, *p* < 0.01), indicating that the negative association between digital inequality and academic success became weaker as perceived institutional support increased.

Likewise, the interaction between institutional support and self-regulated learning was statistically significant (β = 0.12, *p* < 0.05), suggesting that the positive association between self-regulated learning and academic success was stronger among students reporting higher levels of institutional support as detailed in Table 5.

**TABLE 5:** Standardized indirect and interaction effects.

Effect type	Relationship	β	95% CI	p-value
Mediation	Digital Inequality → Self-Regulated Learning → Academic Success	−0.18	[−0.24, −0.12]	<0.001
Moderation	Institutional Support × Digital Inequality → Academic Success	0.15	[0.06, 0.24]	<0.01
Moderation	Institutional Support × Self-Regulated Learning → Academic Success	0.12	[0.02, 0.20]	<0.05

These findings support the theoretical proposition that institutional support functions as a contextual resource that may strengthen positive educational processes while attenuating adverse contextual conditions.

Nevertheless, because moderation effects were estimated from cross-sectional data, these interaction patterns should be interpreted cautiously until replicated using longitudinal designs.

### 3.4 Explained variance

The explanatory capacity of the structural model was evaluated using coefficients of determination (R^2^).

The model explained 29% of the variance in self-regulated learning (R^2^ = 0.29) and 61% of the variance in academic success (R^2^ = 0.61).

According to commonly accepted guidelines for behavioral research, the amount of explained variance observed for academic success can be considered substantial, indicating that the proposed combination of psychological and contextual variables accounted for a considerable proportion of variability in students’ academic outcomes.

Although the reported R^2^ values support the explanatory capacity of the proposed theoretical model, additional indicators such as effect sizes (f^2^) and predictive relevance (Q^2^) would further strengthen the evaluation of model performance and are recommended for future studies.

### 3.5 Summary of findings

Overall, the results were generally consistent with the proposed theoretical framework.

Digital inequality was negatively associated with both self-regulated learning and academic success, whereas self-regulated learning demonstrated the strongest positive association with academic success.

Furthermore, self-regulated learning statistically mediated the association between digital inequality and academic success, while perceived institutional support moderated the relationships between contextual and psychological variables.

Taken together, these findings support the relevance of integrating both individual psychological competencies and contextual educational factors when examining academic success among university students.

However, because the analyses were based on cross-sectional data, the findings should be interpreted as evidence of statistically significant associations rather than causal relationships.

## 4 Discussion

The present study examined the relationships among digital inequality, self-regulated learning, perceived institutional support, and academic success in a sample of Peruvian university students. By integrating psychological and contextual variables within a single structural model, the findings contribute to a more comprehensive understanding of academic success in higher education, particularly in resource-constrained educational environments (Panadero et al., 2017; Pintrich, 2000; Richardson et al., 2012; Sun and Rueda, 2012; Van Laer and Elen, 2017; Zimmerman and Schunk, 2011).

Overall, the results were consistent with the proposed theoretical framework and highlighted the importance of considering both individual learning processes and institutional conditions when examining students’ academic outcomes. Nevertheless, because the study employed a cross-sectional design, these findings should be interpreted as evidence of statistically significant associations rather than causal relationships.

### 4.1 Self-regulated learning and academic success

One of the principal findings was that self-regulated learning showed the strongest positive association with academic success. This finding is consistent with social-cognitive models of self-regulation proposed by Zimmerman (2002) and subsequent empirical reviews indicating that students who effectively regulate their cognition, motivation, and learning behaviors generally report better academic performance.

Rather than viewing self-regulated learning as a fixed personal characteristic, contemporary educational psychology conceptualizes it as a dynamic process that develops through continuous interactions between learners and their educational environments (Schunk and Greene, 2018). Consequently, students’ capacity to self-regulate should be understood as context-dependent and potentially influenced by institutional opportunities, teaching practices, and access to learning resources.

These findings reinforce previous evidence suggesting that promoting self-regulated learning remains one of the most effective educational strategies for improving university students’ academic experiences.

### 4.2 Digital inequality as a multidimensional educational condition

The present findings also indicated that digital inequality was negatively associated with both self-regulated learning and academic success.

These results support previous literature suggesting that digital inequality extends beyond disparities in technological access. Contemporary perspectives emphasize that digital inequality encompasses differences in digital competencies, quality of technology use, and the ability to transform digital resources into meaningful learning opportunities (van Dijk, 2020).

Consequently, students experiencing greater digital inequality may encounter multiple barriers that extend beyond internet connectivity alone. Limited technological resources can restrict opportunities to develop autonomous learning strategies, participate in collaborative learning environments, and effectively engage with digital educational platforms.

Accordingly, digital inequality should be interpreted as a multidimensional educational condition rather than merely a technological limitation.

### 4.3 Self-regulated learning as a statistical mediator

The mediation analyses suggested that self-regulated learning statistically explained part of the association between digital inequality and academic success.

Although mediation models estimated from cross-sectional data cannot establish temporal ordering, these findings are theoretically consistent with educational psychology frameworks proposing that contextual conditions may influence learning outcomes through individual psychological processes.

Rather than interpreting this indirect association as evidence of causality, the present findings should be viewed as supporting a theoretically plausible explanatory pathway that warrants further examination using longitudinal or experimental research designs.

Future studies employing repeated measurements across multiple academic semesters would provide stronger evidence regarding the temporal dynamics underlying these relationships.

### 4.4 Institutional support as a contextual resource

Institutional support also emerged as an important contextual factor associated with academic success.

More importantly, perceived institutional support moderated the associations between digital inequality, self-regulated learning, and academic success, suggesting that supportive educational environments may reduce some of the adverse educational consequences associated with structural disadvantages.

It is important to emphasize that the construct examined in the present study reflects students’ perceptions of institutional support rather than objective institutional characteristics. Therefore, the findings should be interpreted as representing perceived educational experiences rather than institutional quality itself.

This distinction is particularly relevant because students’ subjective perceptions may directly influence motivation, engagement, and learning behaviors regardless of objectively available institutional resources.

### 4.5 Theoretical contributions

The present study contributes to educational psychology in several ways.

First, it integrates psychological and contextual variables within a single theoretical framework, addressing a limitation frequently identified in previous research that has examined these constructs separately.

Second, the study extends existing evidence by examining these relationships in a Latin American public university context, a setting that remains underrepresented in international educational psychology literature.

Third, the findings support contemporary perspectives emphasizing that academic success emerges from continuous interactions between learners and educational environments rather than from isolated psychological or contextual factors.

Nevertheless, alternative theoretical models remain plausible. Reciprocal relationships among digital inequality, self-regulated learning, and institutional support may also exist, and future longitudinal studies should evaluate competing structural models to better understand these complex educational processes.

### 4.6 Practical implications

The present findings have important implications for higher education policy and practice.

Universities should not focus exclusively on expanding technological infrastructure but also invest in interventions that strengthen students’ self-regulated learning competencies.

Programs aimed at improving metacognitive strategies, time management, goal setting, and academic self-monitoring may help students adapt more effectively to digitally mediated learning environments.

Likewise, institutional efforts to improve students’ perceptions of academic support, teaching quality, and technological assistance may contribute to more equitable educational experiences, particularly among students facing digital disadvantages.

## 5 Limitations and future research

Despite the theoretical and empirical contributions of this study, several limitations should be acknowledged when interpreting the findings.

First, the cross-sectional design prevents establishing temporal precedence among the study variables. Although structural equation modeling enabled the simultaneous estimation of direct, indirect, and moderating associations, the observed relationships should not be interpreted as evidence of causality. Future research should employ longitudinal, time-lagged, or experimental designs to examine how self-regulated learning evolves over time and to strengthen causal inference.

Second, the study relied primarily on self-reported measures, including the assessment of academic success. Although self-reported GPA has demonstrated acceptable validity in previous educational research, subjective reporting may introduce social desirability bias, recall bias, and common method variance. Future investigations should incorporate official academic records or institutional databases whenever possible to improve measurement accuracy.

Third, digital inequality was conceptualized as a multidimensional construct; however, additional dimensions such as digital literacy, online learning engagement, digital resilience, and quality of technology-mediated interactions were not directly assessed. Future studies should develop more comprehensive measurement models capable of capturing the complexity of digital inequality in contemporary higher education.

Fourth, institutional support was measured as students’ perceived institutional support rather than objective institutional characteristics. Although perceptions are psychologically meaningful and directly influence educational experiences, future research should combine subjective evaluations with institutional indicators such as technological infrastructure, faculty–student ratios, academic services, and institutional investment in digital education.

Fifth, participants were recruited from a single public university using a convenience sampling strategy. Consequently, caution should be exercised when generalizing these findings to other universities, private institutions, or higher education systems in different countries. Multi-institutional studies employing probabilistic sampling techniques would substantially improve external validity.

Sixth, although the measurement instruments underwent translation, back-translation, and confirmatory factor analysis, additional psychometric procedures such as measurement invariance across demographic groups and broader cross-cultural validation would further strengthen confidence in the instruments’ applicability across diverse educational contexts.

Seventh, the structural model intentionally focused on a limited number of theoretically relevant variables. Nevertheless, other psychological constructs – including academic motivation, self-efficacy, resilience, emotional regulation, psychological well-being, and learning engagement – may also contribute substantially to academic success. Future studies should examine more comprehensive theoretical models incorporating these additional variables.

Finally, self-regulated learning was examined as measured at a single moment in time. Contemporary educational psychology recognizes self-regulated learning as a dynamic and evolving process influenced by continuous interactions between learners and their educational environments. Repeated-measures and longitudinal research designs are therefore needed to better understand how self-regulation develops throughout students’ academic trajectories.

## 6 Conclusion

This study demonstrates that academic success in higher education is shaped by a dynamic interaction between psychological processes and structural conditions. In particular, self-regulated learning emerges as a central psychological mechanism that not only directly predicts academic performance but also mediates the negative effects of digital inequality.

The findings indicate that students who effectively regulate their learning are better equipped to overcome limitations in digital access, highlighting the adaptive function of self-regulated learning in contexts characterized by inequality. At the same time, digital inequality remains a significant barrier, reinforcing the need for systemic interventions that ensure equitable access to technological resources.

Importantly, institutional support plays a critical role in strengthening positive academic outcomes and mitigating the adverse effects of structural disadvantages. Supportive educational environments enhance the benefits of self-regulated learning and contribute to more equitable learning conditions.

By integrating psychological and contextual variables into a unified model, this study advances the field of educational psychology and provides empirical evidence from a Latin American context that remains underrepresented in high-impact research. The results underscore the importance of adopting comprehensive strategies that combine the development of students’ psychological competencies with improvements in institutional support and digital infrastructure.

Ultimately, promoting academic success in higher education requires a multidimensional approach that recognizes the interplay between individual agency and structural opportunity.

## Data Availability

The raw data supporting the conclusions of this article will be made available by the authors, without undue reservation.
